# Multi‐Omics Evidence Based on Spatial Transcriptomics Data Reveals the Therapeutic Value of Copper Death Genes in Glioblastoma

**DOI:** 10.1155/ijog/6453352

**Published:** 2026-01-29

**Authors:** Zhaoliang Xue, Zhengfei Song, Lianjie Mo, Shuxu Yang

**Affiliations:** ^1^ Department of Neurosurgery, SIR Run Run Shaw Hospital, School of Medicine, Zhejiang University, Hangzhou, China, zju.edu.cn

**Keywords:** cuprotosis, hsa-miR-93-5p, low-grade glioma, risk score

## Abstract

**Background:**

Cuprotosis is an emerging form of copper‐dependent programmed cell death, while low‐grade gliomas (LGGs) represent a common subtype of primary brain tumors.

**Methods:**

Datasets from The Cancer Genome Atlas and TargetScan were utilized to identify cuprotosis‐related microRNAs (CRMs). Univariate Cox and Lasso regression analyses identified CRMs linked to prognostic outcomes. Prognostic profiles for patients with LGG were constructed using multivariate Cox regression and validated for risk stratification in the CGGA external validation cohort. The study examined clinical features, mutational status, immune cell infiltration, signaling pathways, and immune checkpoint expression across different risk groups. Functional experiments assessed the biological significance of key model genes.

**Results:**

Seven CRMs significantly associated with LGG prognosis were identified. The correlation between the CRM signature and poor prognosis in high‐risk LGG cases was validated through Kaplan–Meier survival analysis, yielding a one‐year area under the curve (AUC) of 0.849, indicating strong predictive accuracy. Risk scores were linked to 1p/19q co‐deletion, IDH mutation, and tumor grade, with the model outperforming traditional clinicopathological criteria. Molecular enrichment analyses, including Gene Set Enrichment Analysis (GSEA) and Gene Set Variation Analysis (GSVA), revealed significant associations between high‐risk subgroups and pathways related to tumorigenesis and immune dysregulation. Increased tumor mutational burden and elevated IC expression were noted in high‐risk cohorts. Furthermore, miR‐93‐5p was validated as a critical gene, with its disruption leading to significant reductions in GBM cell proliferation, migration, and invasion.

**Conclusion:**

The novel CRM signature enhances the prognostic landscape for patients with LGG, offering a new framework for evaluating immunotherapeutic efficacy.

## 1. Introduction

Gliomas, which arise from glial cells or their progenitors, are a prevalent type of primary brain tumor distinguished by marked heterogeneity and a tendency to induce severe comatose states in patients [1]. Low‐grade gliomas (LGGs), a specific glioma subset, are defined by their diffuse infiltrative growth and gradual progression, which together give rise to unique clinicopathological manifestations. A striking feature of these tumors is their extensive genetic and transcriptional heterogeneity, with each neoplasm potentially exhibiting unique gene expression profiles [2]. The field of glioma research has been significantly advanced by next‐generation genomic sequencing technologies, which have unveiled a spectrum of critical genetic alterations, including mutations in IDH1/2, TP53, and the loss of ATRX. These genetic events not only drive the oncogenic process but are also closely associated with tumor grading, prognostic outcomes, and responses to therapy. The TCGA Research Network has conducted extensive genomic analyses that have refined glioma classification, expanding the definition of “low‐grade glioma” to encompass both Grade 2 and Grade 3 tumors, thereby reflecting a more nuanced understanding of their molecular landscape [3]. In contrast to high‐grade gliomas, LGGs typically follow a more indolent clinical course and are associated with extended patient survival. However, they possess an inherent capacity for spontaneous malignant transformation into high‐grade gliomas, a transition that often results in a significant reduction in patient survival duration [4]. Advanced genomic analyses suggest that this malignant transformation may be associated with the progressive accumulation of specific genetic mutations, identifying potential molecular targets for early therapeutic intervention. In the context of glioma classification and diagnosis, an innovative approach has emerged that is based on specific genetic markers. Notably, the co‐deletion of chromosome arms 1p/19q and IDH mutations have been identified as salient genetic markers that enable the robust differentiation of glioma subtypes and provide a molecular framework for personalized treatment strategies [5, 6]. Surgical resection remains the primary treatment modality for LGGs, although its feasibility is often constrained by factors such as tumor location, patient comorbidities, and surgical risks. For patients who are not candidates for surgery, radiotherapy and chemotherapy serve as essential alternative treatment options [7]. Despite the use of multimodal therapeutic approaches, LGG lesions may persist in growth and recurrence, underscoring the need for the development of novel therapeutic strategies to enhance patient outcomes.

The dysregulation of iron metabolism has emerged as a critical factor implicated in carcinogenesis. Ferroptosis, a regulated form of cell death that is dependent on iron metabolism, has garnered significant research attention. Its molecular mechanism is centered around disrupted intracellular iron homeostasis, which leads to excessive production of reactive oxygen species (ROS) and lipid peroxidation, ultimately resulting in cell membrane disruption and cellular death [8]. Recent scholarly investigations have also illuminated the analogous role of copper ions in mediating cell death. Cuprotosis, a novel form of cell death induced by copper ions, is initiated through the interaction of copper ions with lipid‐acylated components of the tricarboxylic acid (TCA) cycle. This molecular interaction triggers the aberrant aggregation of lipid‐acylated proteins and the concomitant loss of iron–sulfur cluster proteins, thereby instigating a proteotoxic stress response that culminates in cell death [9]. Studies have revealed that the serum and tumor tissue copper levels of patients with cancer are significantly higher than those of healthy patients [10, 11]. Moreover, preclinical studies suggested that copper ion metal carriers may be used to kill cancer cells [12]. Consequently, various copper ion carriers and chelators have been actively investigated in preclinical anticancer studies, demonstrating preliminary efficacy [13, 14].

MiRNAs, which are endogenously synthesized noncoding single‐stranded RNA molecules of approximately 22 nucleotides in length, exert significant regulatory effects on post‐transcriptional gene expression by binding to the 3 ^′^ untranslated regions (3 ^′^ UTRs) of target mRNAs, thereby inhibiting translation or inducing mRNA degradation [15]. In addition to gene regulation, dysregulation of miRNA function has been associated with many diseases, including cancer. miRNAs are involved in various biological regulatory processes and are associated with tumorigenesis, progression, and metastasis [16]. Moreover, miRNAs have been recognized to play a pivotal role in modulating tumor metabolism by regulating the expression of genes associated with metabolic processes, thereby orchestrating the energy metabolic reprogramming that underpins the rapid proliferation and invasive potential of cancer cells [17].

In the conduct of this study, we undertook the identification of differentially expressed circular RNAs (CRMs) associated with LGGs and employed them to construct a predictive model designed for patient risk stratification. Through the application of risk score algorithms, patients were categorized into high‐risk and low‐risk cohorts to facilitate the evaluation of their respective clinical characteristics. Validation experiments revealed that miR‐93‐5p, a miRNA of critical importance in glioma biology, significantly attenuated the proliferative, migratory, and invasive capacities of glioblastoma cells when its expression was experimentally disrupted. This preclinical evidence suggests that miR‐93‐5p may represent a promising therapeutic target for improving the prognostic outlook of patients afflicted with LGGs.

## 2. Materials and Methods

### 2.1. Acquisition of Raw Data

We downloaded miRNA sequencing information, mutation data, and clinicopathological characteristics of the TCGA‐LGG cohort from the UCSC Xena website. The miRNA expression profiles and clinical data of patients with LGG in the externally validated risk model were obtained from the CGGA data portal, with 139 LGG tissue samples in the CGGA cohort. In addition, we performed further validation using the GSE138764. Detailed clinical characteristics of patients in the TCGA and CGGA databases are summarized in Supplementary Table S1. Based on the Targetscan database, we predicted CRG‐targeted regulatory miRNAs [18]. Supplementary Table S2 shows the CRGs and predicted miRNAs.

### 2.2. Mutation Landscape Between Different Risk Subgroups

The “oncoplot” function of the R software’s “maftools” package was used to create two waterfall plots to examine the specific mutation characteristics that differentiate the high‐risk and low‐risk groups.

### 2.3. Nomogram and Calibration

We created Nomograms for 1‐year, 3‐year, and 5‐year overall survival (OS) for patients with LGG by combining risk scores with age, gender, race, tumor stage, IDH mutation status, and 1p/19q deletion. The Hosmer–Lemeshow test calibration curve was used to show the degree of agreement between the actual and typical expected outcomes.

### 2.4. Cell Culturing

All human glioma cell lines utilized in this study (NHA, U87, LN229, and LN18) were authenticated using the STR method. Cells were cultured in RPMI1640 medium supplemented with 10% fetal bovine serum (FBS), and maintained at a constant temperature of 37°C.

### 2.5. Cell Transfection

The miR‐93‐5p inhibitor was introduced into glioma cells at a final concentration of 10 *μ*M. The sequence of the miR‐93‐5p inhibitor was 5 ^′^‐CUACCUGCACGAACAGCACUUUG‐3 ^′^. Cells were transfected in DMEM lacking FBS for 6 h, after which the medium was replaced with FBS‐containing medium. Transfected cells were then maintained for subsequent analyses.

### 2.6. RT‐PCR Assay

Total RNA isolation and extraction were performed using RNAiso‐Plus (Takara), as recommended by the manufacturer. Subsequently, RNA was reverse‐transcribed into cDNA with the aid of a high‐capacity gene synthesis kit (Takara, China). Optimal reaction conditions were established based on predetermined criteria. U6 was selected as an internal reference for abundance assessment, and relative expression levels were quantified using the 2^−*ΔΔ*Ct^ method. The primer sequences for miR‐93‐5p were F: 5 ^′^‐CAAAGUGCUGUUCGCAGGU AG‐3 ^′^ and R: 5 ^′^‐CUACCUGCACGAACAGCACUUUG‐3 ^′^. For U6, the primers were F: GCTCGCTTCGGCAGCACA and R: GAACGCTTCACGAATTTGCGTG.

### 2.7. Cell Viability Assay

Cell viability was assessed using the Cell Counting Kit‐8 (CCK8). Glioma cells were seeded in 96‐well plates. Optical density at 450 nm was measured using a microplate reader. Each experimental condition was performed in triplicate, and each experiment was repeated three times.

### 2.8. Statistical Analysis

All analyses were conducted using R version 4.1.1 and its support packages. In all statistical analyses, a *p* value of 0.05 was considered statistically significant.

## 3. Results

### 3.1. Single‐Cell and Spatial Transcriptomic Data Reveal the Expression of Copper Death‐Related Genes in the Glioblastoma Microenvironment

Firstly, the glioblastoma samples from the single‐cell dataset GSE173278 were analyzed. After quality control and clustering, six cell subpopulations were successfully identified (Figure 1a), namely Astrocyte, Cancer (neoplastic), Myeoid, Neuron, OPC, Oligodendrocyte, and Vascular. A comparison of the G2M score across different subpopulations revealed that tumor cells are more frequently in the G2/M phase (Figure 1b). An expression comparison of copper death‐related genes across various subpopulations showed differences in expression levels, with the most significant expression in tumor and neuron cells. This indicates that, from a biological developmental perspective, copper death‐related genes are crucial in the formation of glioblastoma or LGG. During this process, the genes HFE2L2, DLD, and GSCH were more highly expressed in the current dataset, predominantly in tumor and neuronal cells. The interaction between these two may be a solution to overcoming the survival challenges of glioblastoma in the future (Figures 1g, 1h, and 1i). Further attempts were made to substantiate this result through spatial transcriptomic evidence, showing that the copper death gene set is concentrated in tumor regions and most closely associated with functions such as the IL6 pathway in the hallmarks gene set (Figures 1j, 1k, 1l, 1m, 1n, and 1o).

Figure 1(a) UMAP plot showing cell clustering of GSE173278. The clusters are divided into six cell populations: astrocyte, cancer (neoplastic), myeoid, neuron, OPC, oligodendrocyte, and vascular. (b) Heatmap showing the expression of cell cycle‐related genes across different cell populations. (c) Bubble plot showing the expression of copper death genes across different cell populations. (d–i) UMAP plots and violin plots showing the expression of HFE2L2, DLD, GSCH in the copper death single‐cell dataset. (j) Spatial transcriptomic profile showing cell area clustering and division. (k) Spatial heat map showing high expression in tumor areas. (l) Bar chart comparing the expression content of tumor cells across different spatial clusters. (m) Spatial heat map of copper death‐related genes expression. (n) Spatial heat map indicating the activation of the IL6‐related pathway. (o) Bar chart comparing the expression annotation of all cells across different spatial clusters.  ^∗^ *p* < 0.05,  ^∗∗^ *p* < 0.01,  ^∗∗∗^ *p* < 0.001.

**Figure:**
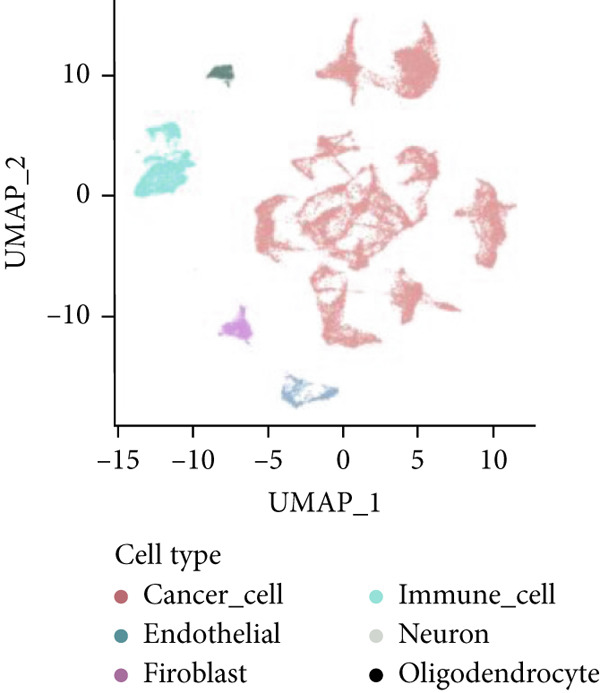
(a)

**Figure:**
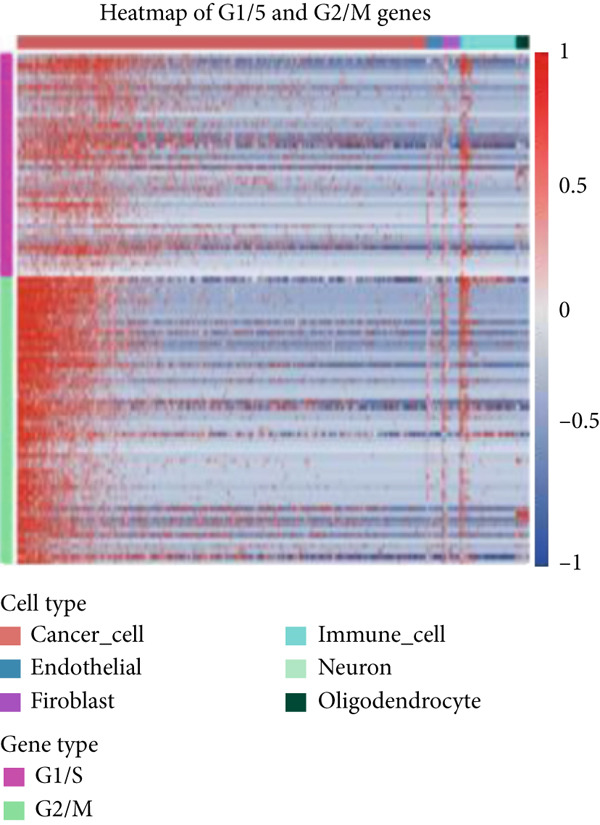
(b)

**Figure:**
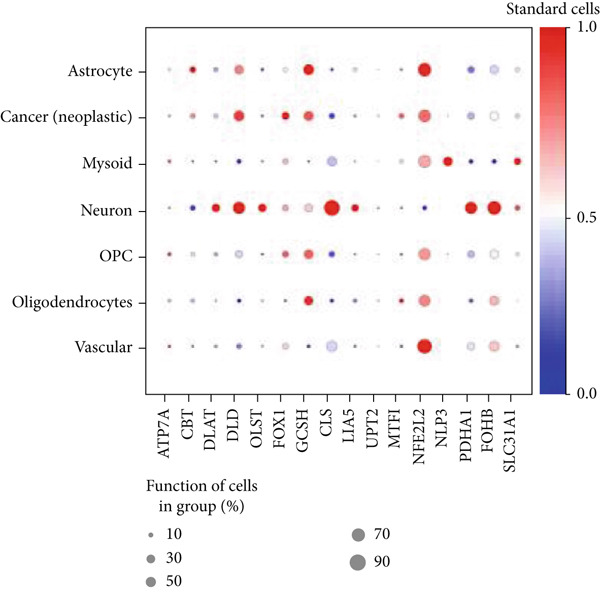
(c)

**Figure:**
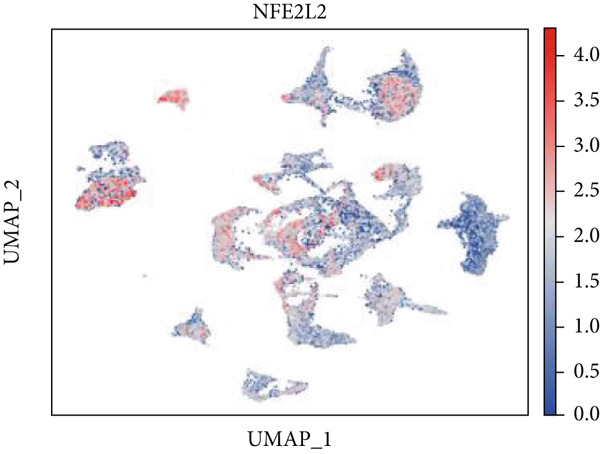
(d)

**Figure:**
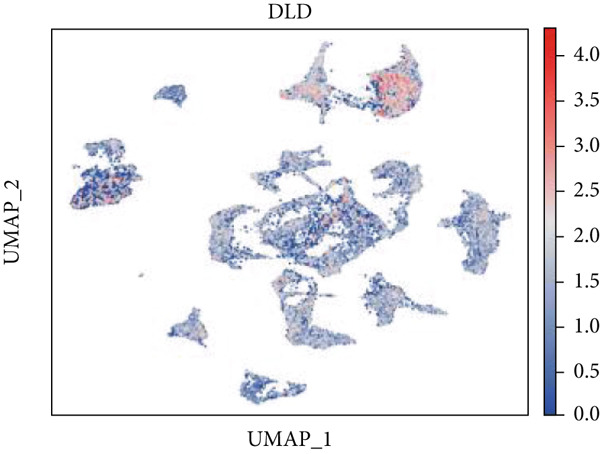
(e)

**Figure:**
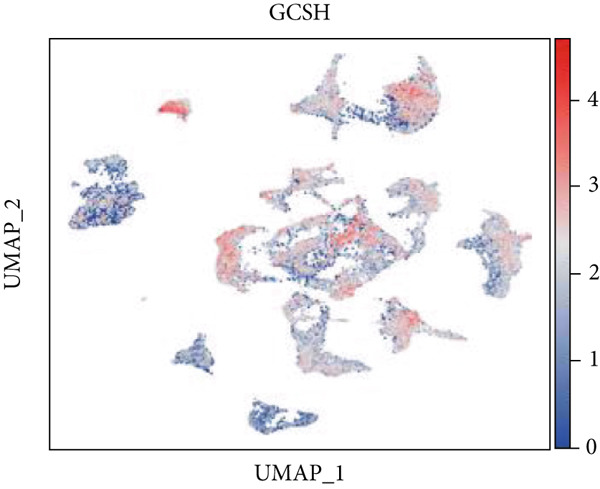
(f)

**Figure:**
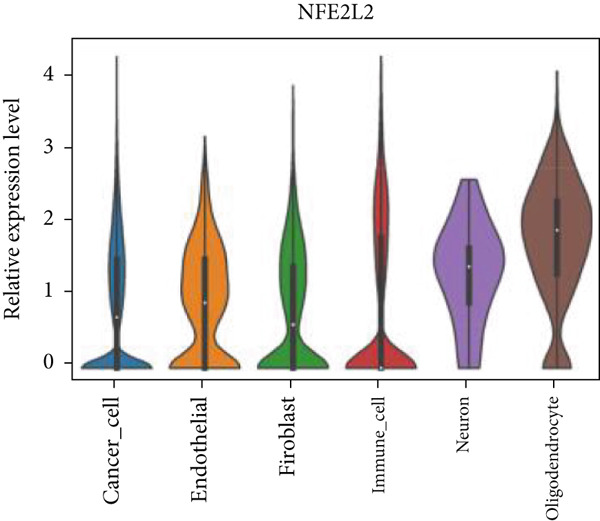
(g)

**Figure:**
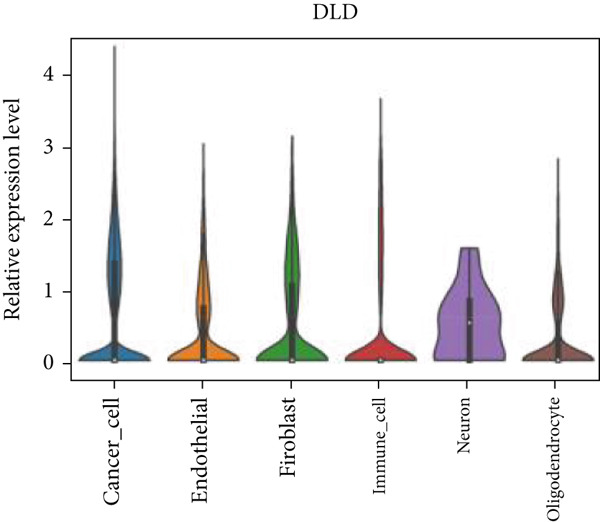
(h)

**Figure:**
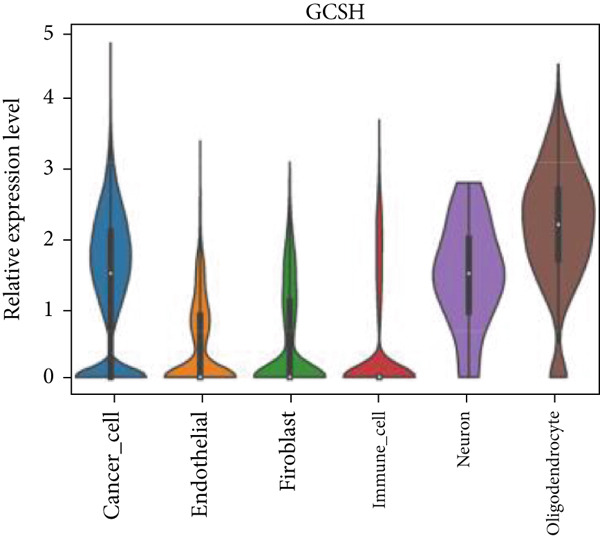
(i)

**Figure:**
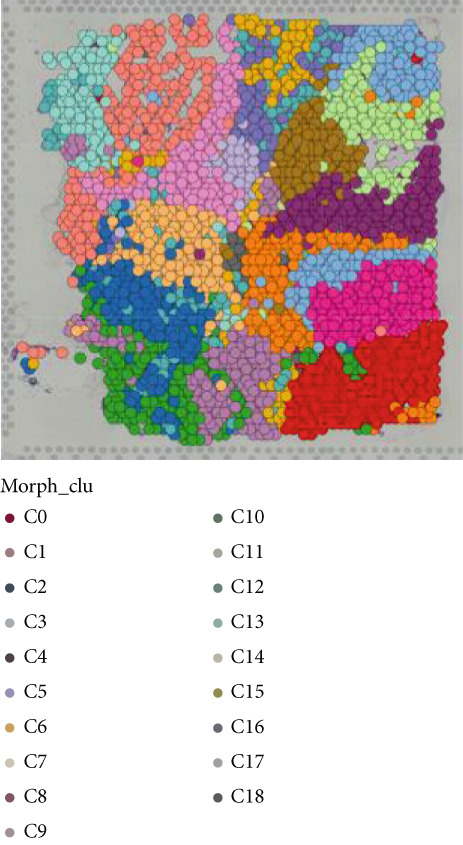
(j)

**Figure:**
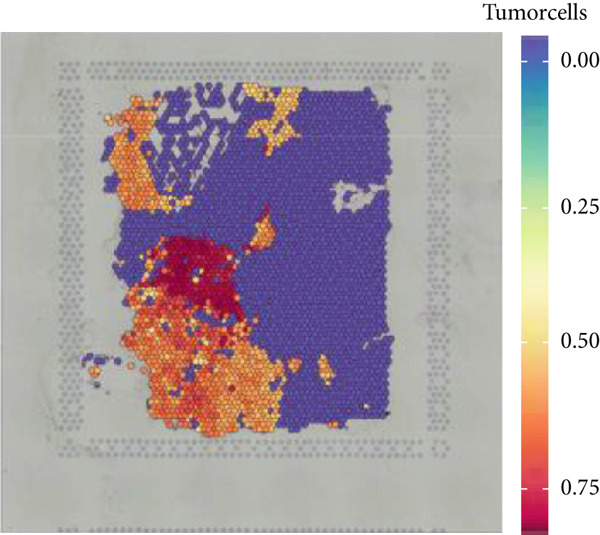
(k)

**Figure:**
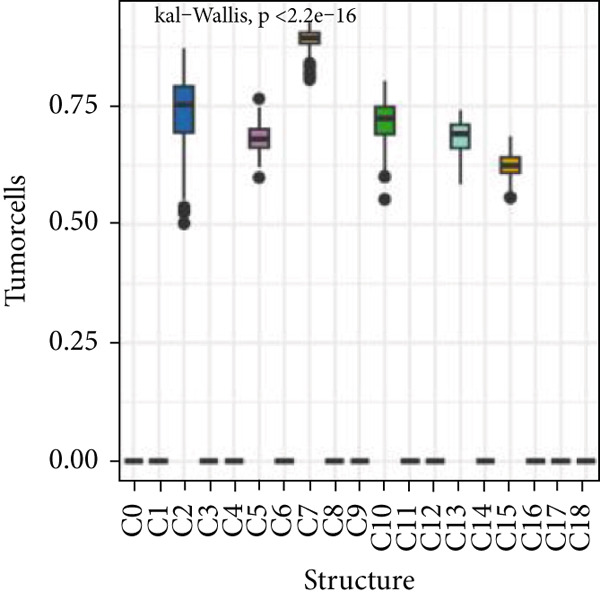
(l)

**Figure:**
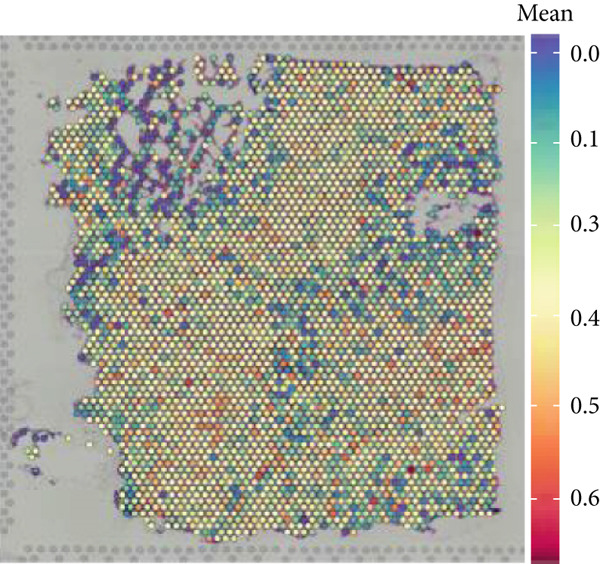
(m)

**Figure:**
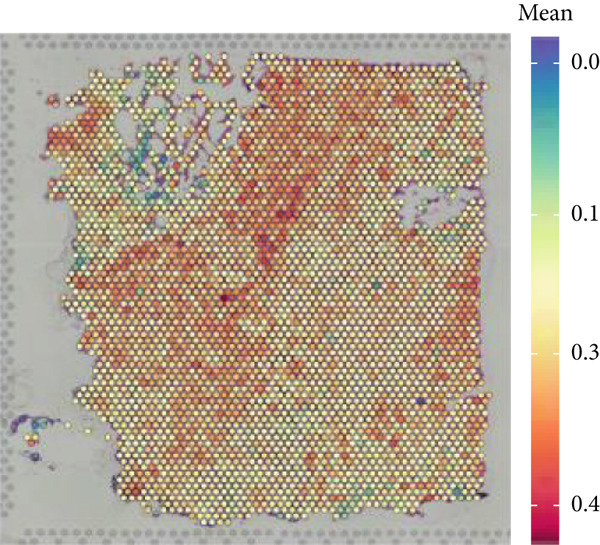
(n)

**Figure:**
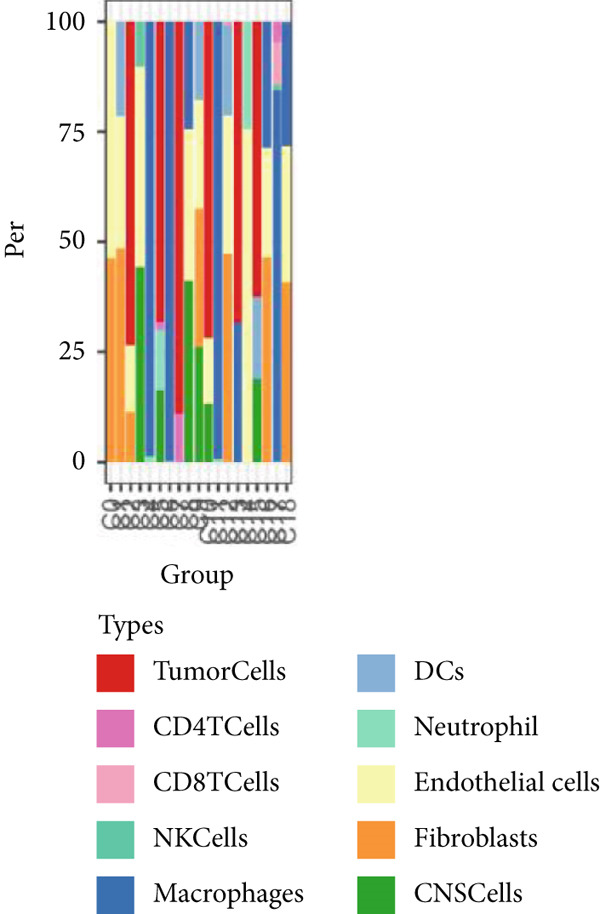
(o)

### 3.2. Identification of Prognostic miRNAs in Patients With LGG

Through the Targetscan webpage, we predicted the miRNAs targeting the regulation of these genes based on the reported cuprotosis‐related genes (CRGs). Figure 2a shows the Sankey diagram of cuprotosis mRNA and miRNA interconnections. Next, we performed a differential analysis of tumor and normal tissues in the TCGA‐LGG cohort and identified 157 differential miRNAs. Sixty‐three miRNAs were upregulated and 94 miRNAs were down‐regulated in tumor tissues (Figure 2b). CRMs were then predicted and the differential genes were selected (Figure 2c). In order to create an expression matrix for additional investigation, 43 miRNAs were chosen. To determine possible predictive markers for LGG in the TCGA population, we lastly conducted a univariate Cox regression analysis. A total of 16 miRNAs were shown to be prognostic indicators (Figure 2d).

Figure 2Prognosis‐associated miRNAs in LGG identified using differential analysis and Univariate Cox analysis. (a) Correspondence between miRNAs and their target‐bound cuprotosis‐related mRNAs. (b) The expression difference of miRNAs in the TCGA cohort. (c) Venn diagram between differential miRNA and miRNA targeting cuprotosis‐related mRNAs. (d) Univariate Cox analysis was performed on 43 intersecting miRNAs, and 16 prognostic miRNAs were obtained.

**Figure:**
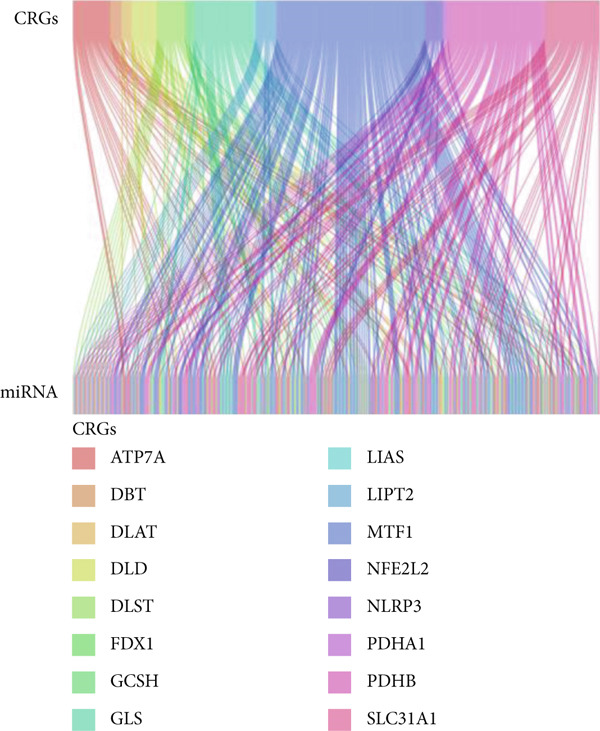
(a)

**Figure:**
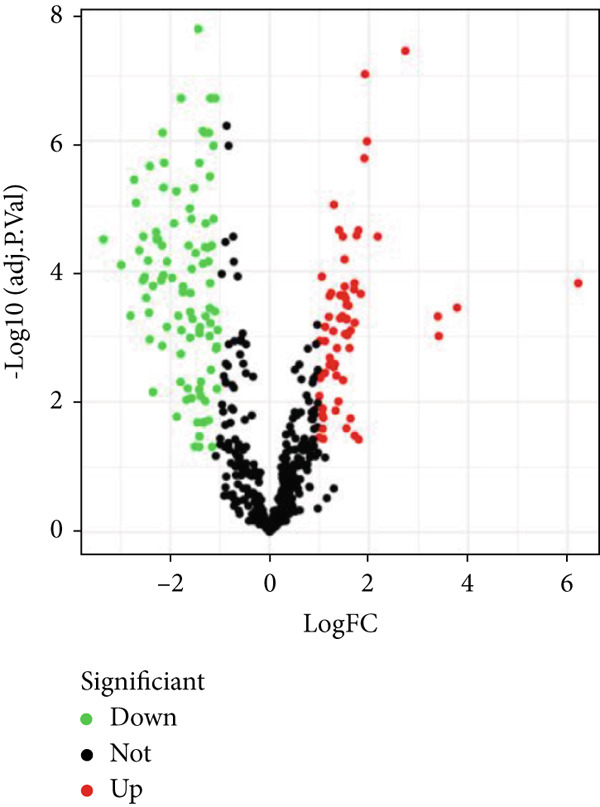
(b)

**Figure:**
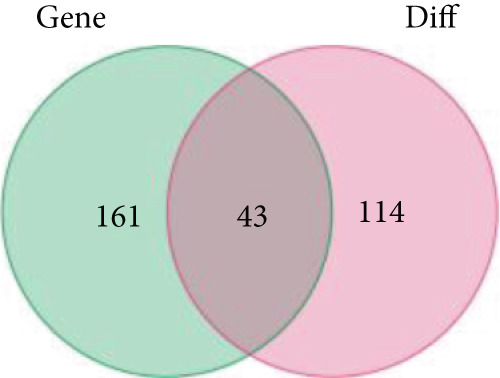
(c)

**Figure:**
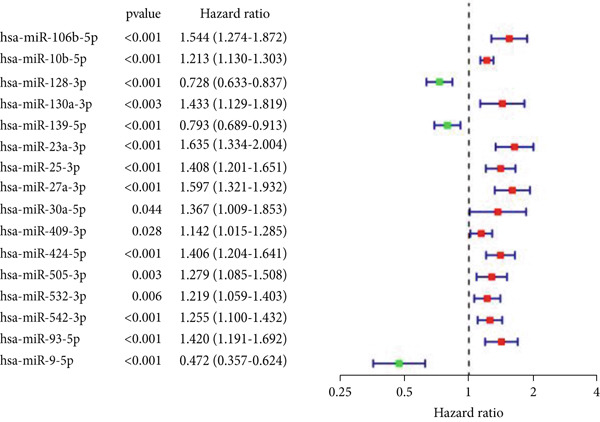
(d)

### 3.3. Construction of the CRM Signature

We used LASSO regression analysis to identify 10 miRNAs on 16 prognostic miRNAs, prevent overfitting, and enhance the precision and interpretability of prognostic features (Figure 3a,b). In the end, multivariate Cox analysis indicated that seven miRNAs, including hsa‐miR‐10b‐5p, hsa‐miR‐128‐3p, hsa‐miR‐27a‐3p, hsa‐miR‐30a‐5p, hsa‐miR‐409‐3p, hsa‐miR‐424‐5p, and hsa‐miR‐93‐5p, were independent prognostic factor. In Supplementary Table S3, the correlation coefficients are displayed. Patients were then divided into high‐risk and low‐risk groups based on the risk score’s median value. Patients in the high‐risk group had a worse OS than those in the low‐risk group, according to survival curves (Figure 3c,d). Heat maps revealed variations in the expression of the seven miRNAs in the model in the risk group, and risk plots further highlighted specific survival outcomes (Figure 3e,f) and CGGA external validation cohort (Figure 3g,h). Additionally, it was demonstrated that for these patients in the TCGA cohort, the risk score functioned well in predicting OS (AUCs for 1, 3, and 5‐year OS: 0.849, 0.792, and 0.735; Figure 3i). Similar results were observed in the CGGA cohort (Figure 3j). The PCA and t‐SNE analyses for the TCGA and CGGA cohorts are displayed in Figure 4. According to Figure 4a,b, the TCGA cohort’s patients were distributed differently according to their risk category. According to Figure 4c,d, the patients in the CGGA cohort who belonged to various risk categories were distributed in two distinct ways. In both PCA and t‐SNE analyses, the two risk groups scarcely crossed paths, showing the viability of utilizing the aforementioned 7‐miRNA signature.

Figure 3Creation and verification of prognostic models for patients with LGG. (a, b) Eight DEGs were chosen for multivariate analysis based on the outcomes of lasso regression analysis. (c, d) Survival curves used to assess the TCGA and CGGA cohorts’ capacity for risk categorization. (e, f) The survival status of each sample in the TCGA and CGGA cohorts was depicted using risk plots. (g, h) Heat map showing how each gene in the risk group expresses itself differently. (i, j) The built risk models’ ability to predict outcomes for the TCGA and CGGA cohorts was assessed using ROC curves.

**Figure:**
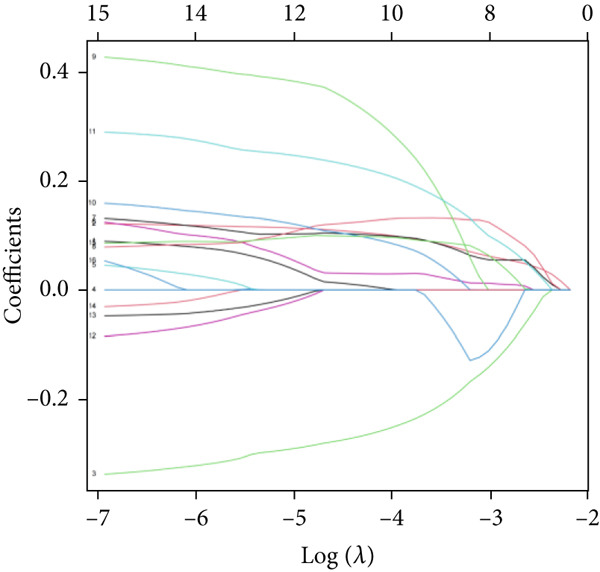
(a)

**Figure:**
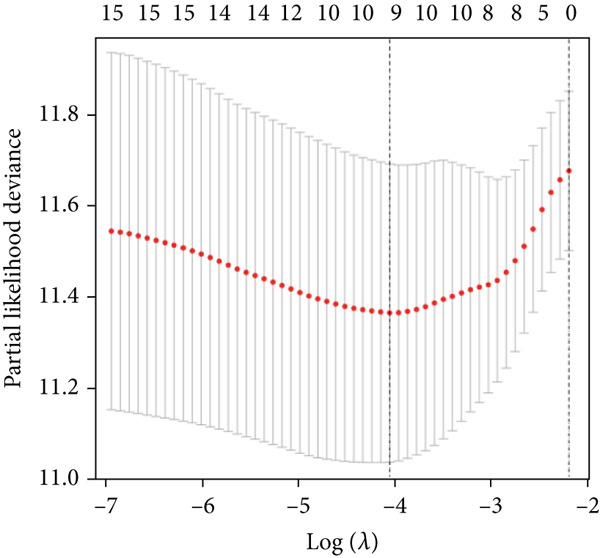
(b)

**Figure:**
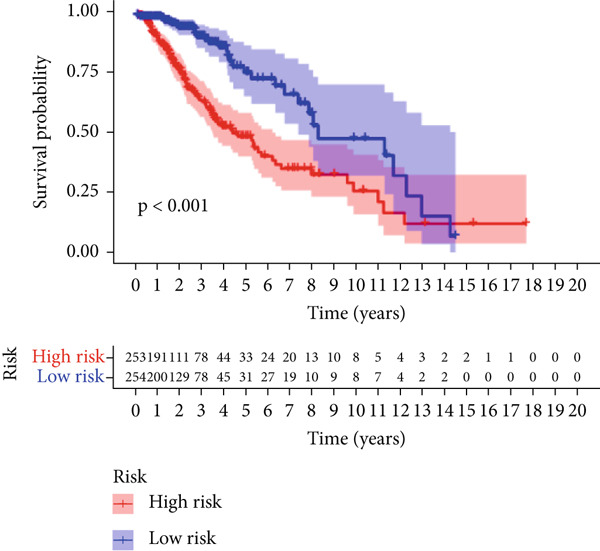
(c)

**Figure:**
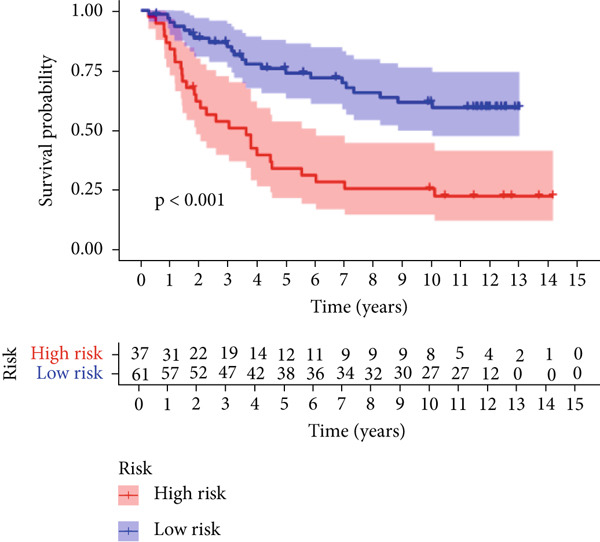
(d)

**Figure:**
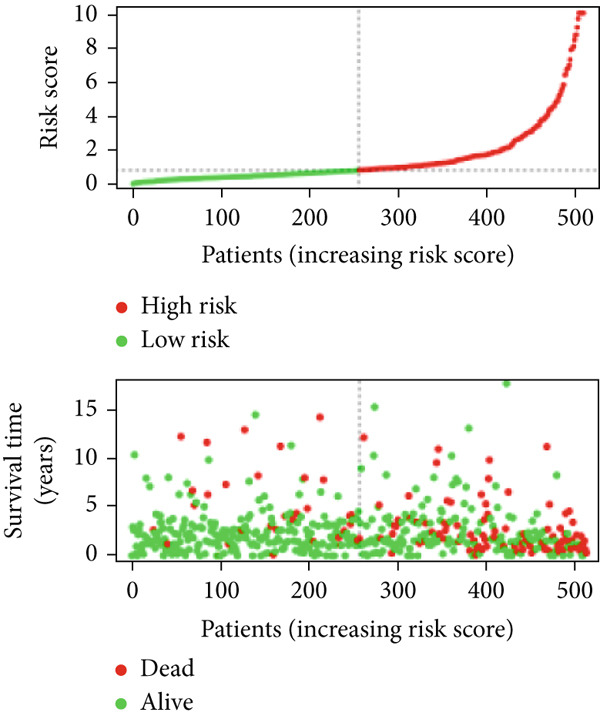
(e)

**Figure:**
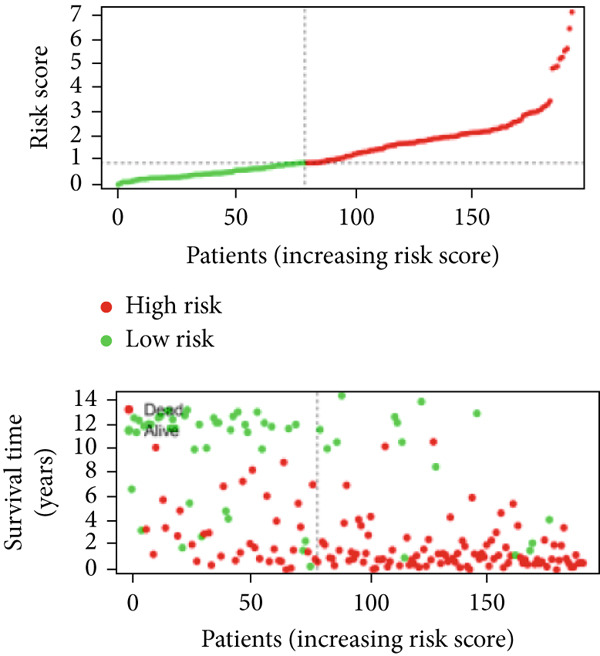
(f)

**Figure:**
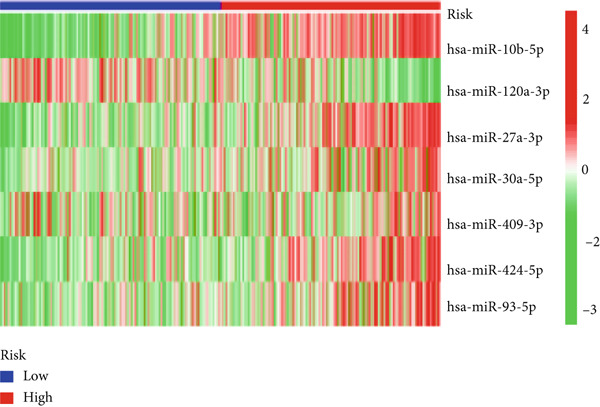
(g)

**Figure:**
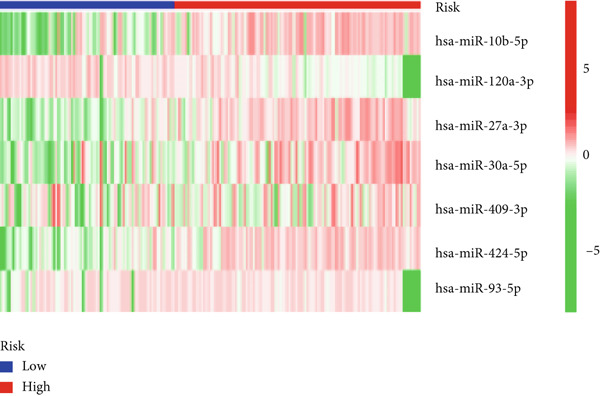
(h)

**Figure:**
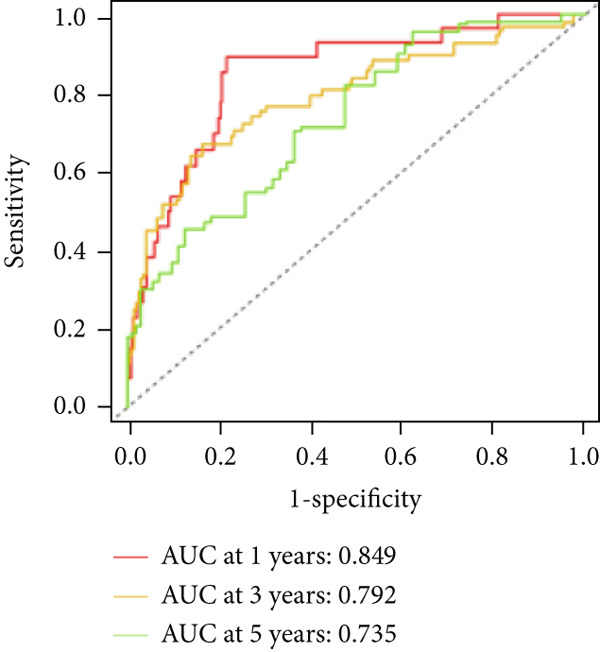
(i)

**Figure:**
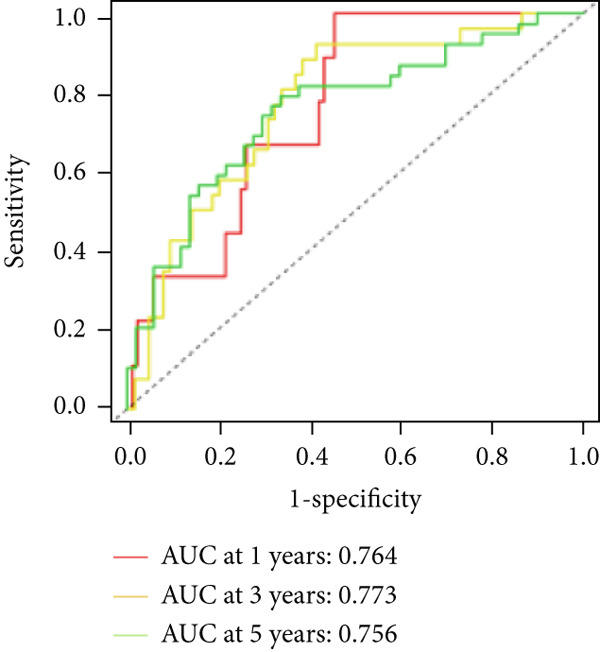
(j)

Figure 4PCA plot and t‐SNE analysis in both TCGA and CGGA cohorts. (a) PCA plot in TCGA cohort. (b) t‐SNE analysis in TCGA cohort. (c) PCA plot in CGGA cohort. (d) t‐SNE analysis in CGGA cohort.

**Figure:**
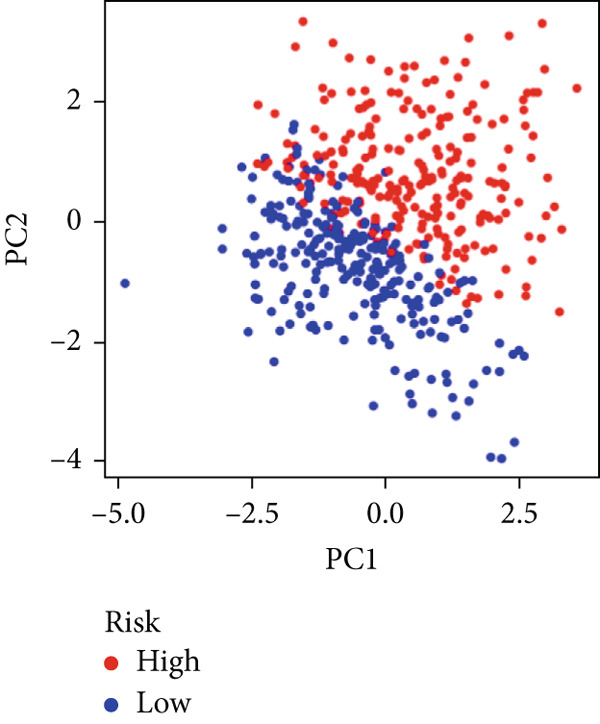
(a)

**Figure:**
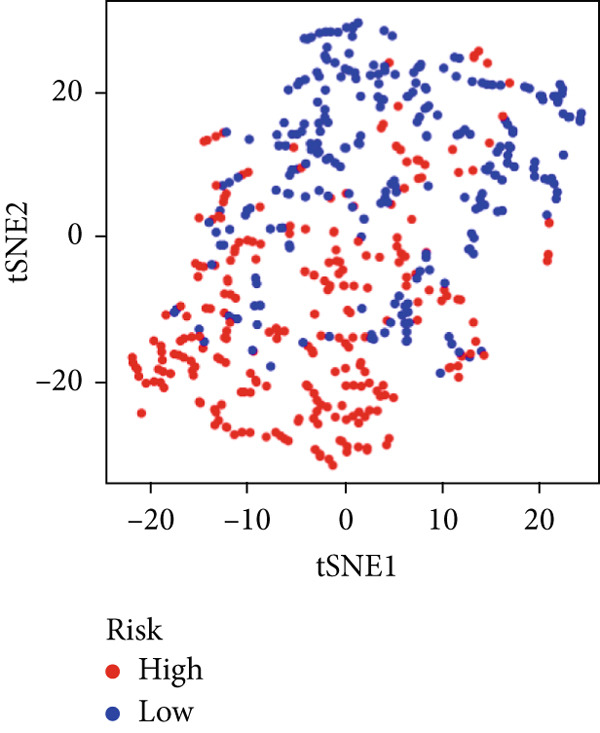
(b)

**Figure:**
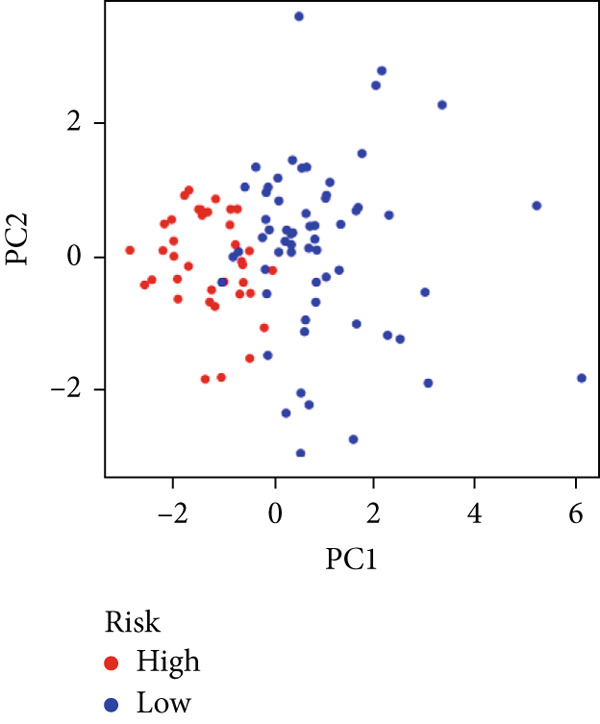
(c)

**Figure:**
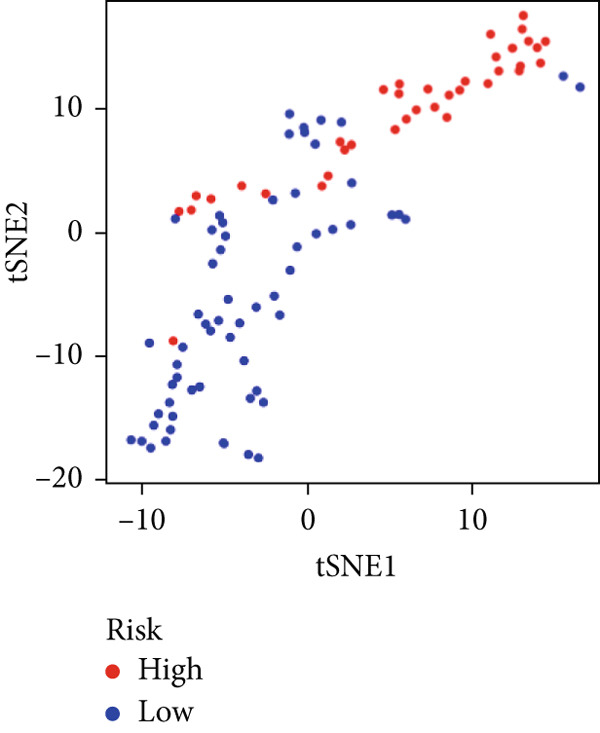
(d)

### 3.4. Establishment of a Prognostic Nomogram and Clinical Features

The TCGA cohort’s univariate and multivariate Cox analysis revealed that risk score was an independent predictive predictor for LGG (Figure 5a,b). Age, tumor grade, IDH mutation status, and risk grouping were all taken into account while creating the nomogram based on the findings of the multivariate cox analysis of the TCGA cohort (Figure 5c). Calibration plots showed that the predictions made by the nomogram were accurate (Figure 5d). The risk score’s area under the curve (AUC) was greater than those of other clinicopathological characteristics (Figure 5e). The findings imply the reliability of the predictive characteristics of the 7‐miRNA signature. The bar graph also demonstrated the relationship between the risk score and the tumor’s histological type, tumor grade, IDH mutation status, and 1/19q deletion status (Figure 5f). Based on the above observations, we found that individuals who died, received radiotherapy, had G3 staging, had no mutations in IDH, had no co‐deficiency in 1p/19q, and had astrocytoma tissue type exhibited higher risk scores (Figure 6, *p* < 0.001). To study the connection between risk factors and prognosis, patients with TCGA‐LGG were divided into groups according to age, gender, tumor grade, histological type, race, IDH mutation status, and 1p/19q co‐deletion. Patients in the TCGA cohort’s low‐risk group had considerably longer OS than those in the high‐risk group for various stages (Figures 7a, 7b, 7c, 7d, 7e, 7f, and 7g). The favorable prognosis of patients in these subgroups and the small number of patients may be the causes of the subgroup variations in IDH mutation, 1p/19q co‐deletion, and non‐Caucasian subgroup outcomes. These findings imply that the prognosis of patients with LGG of various ages, genders, and characteristics like IDH status may also be predicted using the 7‐miRNA signature.

Figure 5Prognostic value of risk scores in patients with LGG. (a) Univariate and (b) multivariate COX analysis for evaluating the prognostic signature and clinical features (including age, race, gender, IDH state, and 1p/19q codeletion). (c) Nomogram of risk groupings and clinical characteristics predicting survival at 1, 3, and 5 years. (d) Calibration curves tested the agreement between actual and predicted outcomes at 1, 3, and 5 years. (e) The AUC values of the prognostic signature and clinical features. (f) Bar chart of clinical characteristics associated with risk scores.  ^∗^ *p* < 0.05,  ^∗∗^ *p* < 0.01,  ^∗∗∗^ *p* < 0.001.

**Figure:**
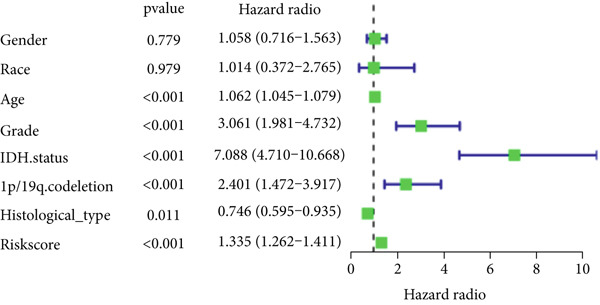
(a)

**Figure:**
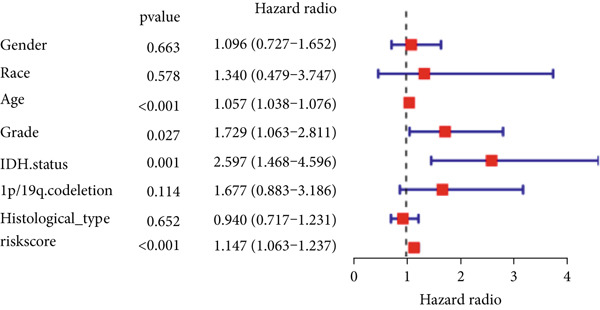
(b)

**Figure:**
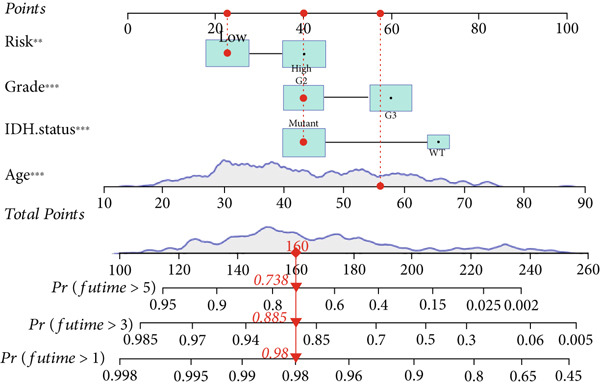
(c)

**Figure:**
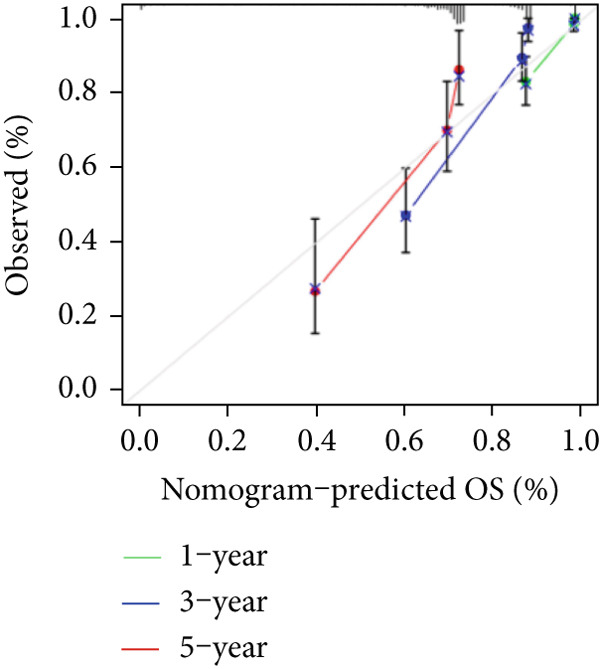
(d)

**Figure:**
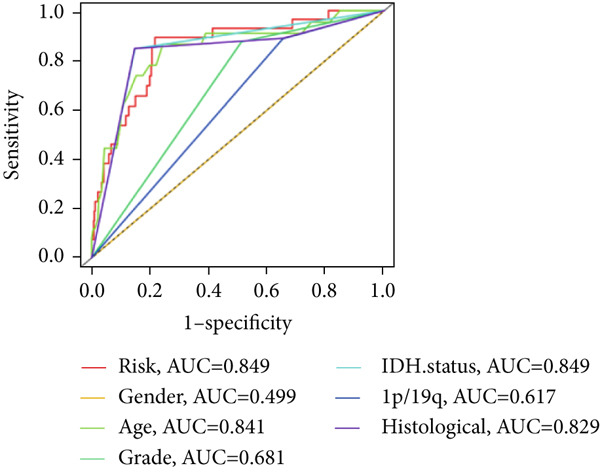
(e)

**Figure:**
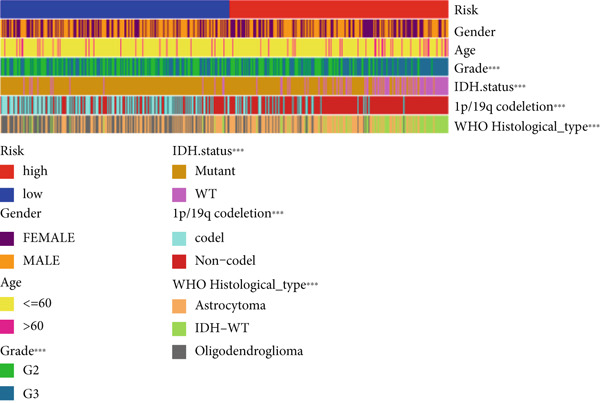
(f)

Figure 6Variations in risk scores among the TCGA cohort’s various clinical characteristic groupings. (a) Survival outcome, (b) radiotherapy, (c) grade, (d) IDH mutation status, (e) 1p/19q. codeletion status and (f) histological type.  ^∗^ *p* < 0.05,  ^∗∗^ *p* < 0.01,  ^∗∗∗^ *p* < 0.001.

**Figure:**
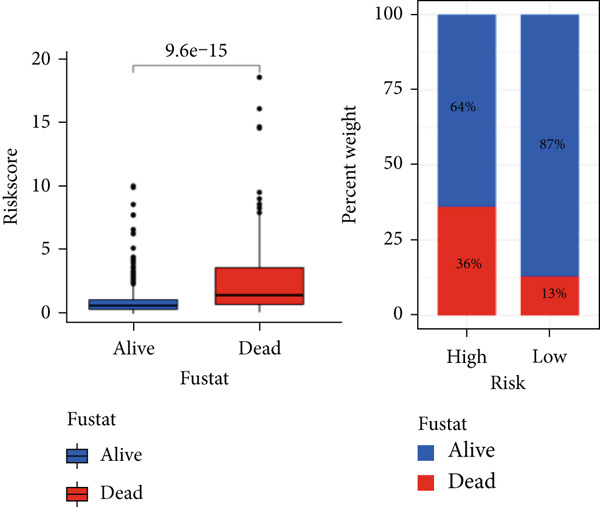
(a)

**Figure:**
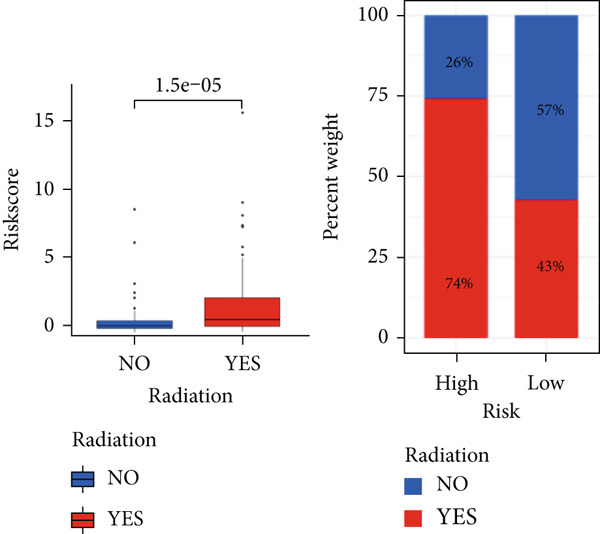
(b)

**Figure:**
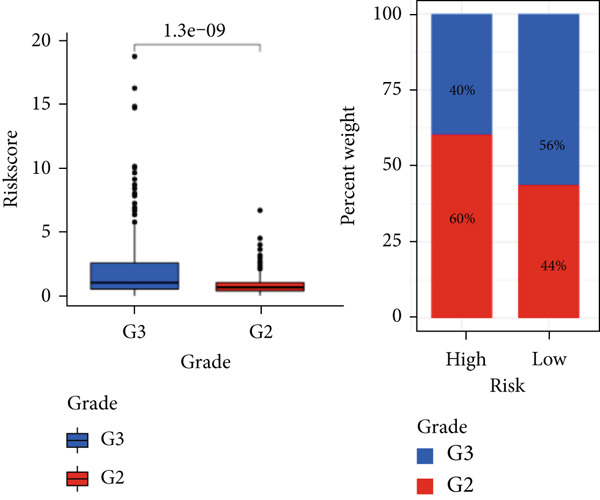
(c)

**Figure:**
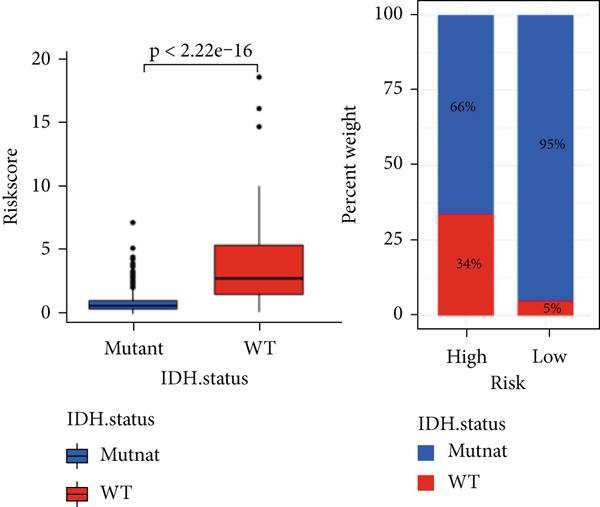
(d)

**Figure:**
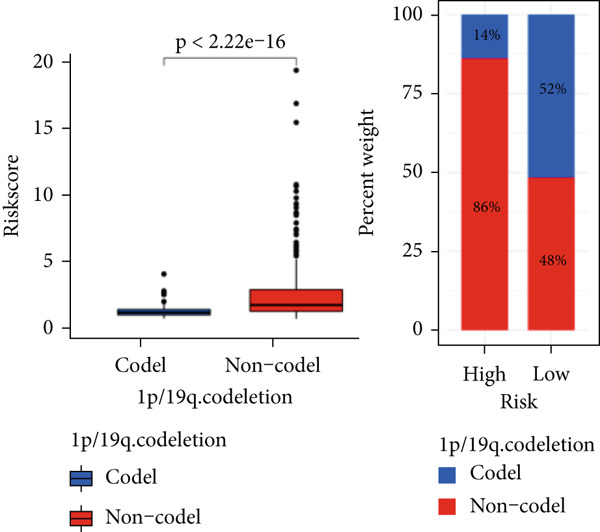
(e)

**Figure:**
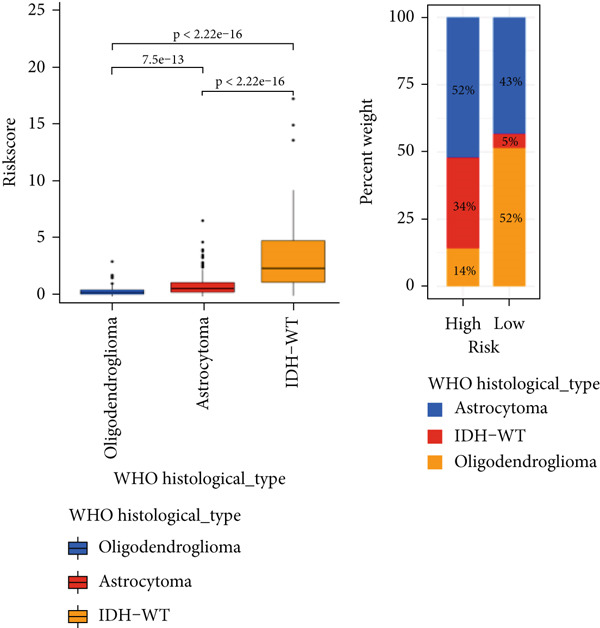
(f)

Figure 7Kaplan–Meier survival curves for the low‐risk and high‐risk groups in the TCGA cohort sorted by different clinicopathological variables. (a) Age, (b) sex, (c) grade, (d) race, (e) IDH mutation status, (f) 1p/19q.codeletion, and (g) histological type.

**Figure:**
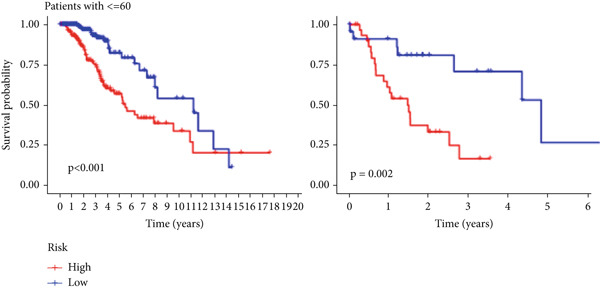
(a)

**Figure:**
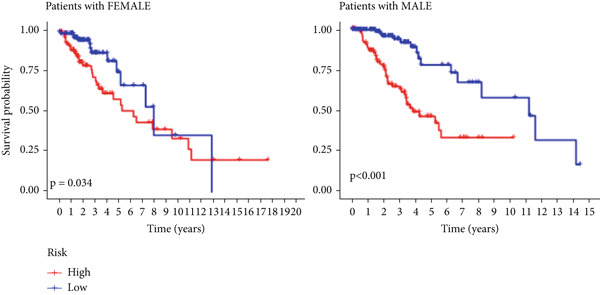
(b)

**Figure:**
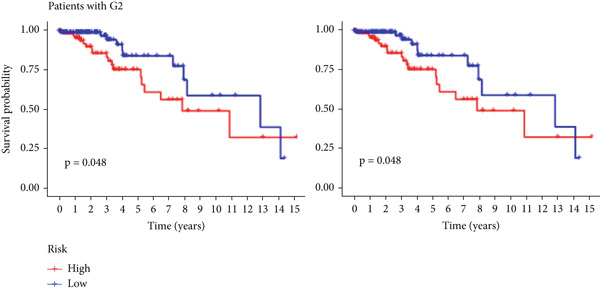
(c)

**Figure:**
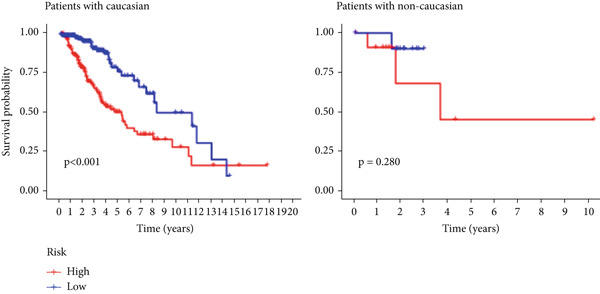
(d)

**Figure:**
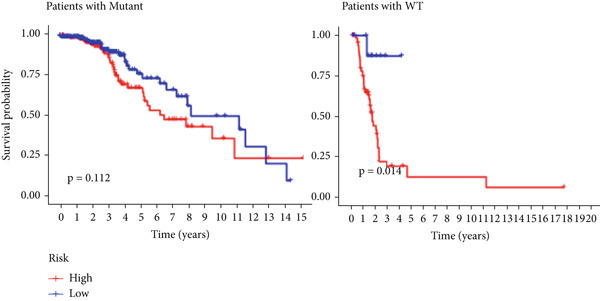
(e)

**Figure:**
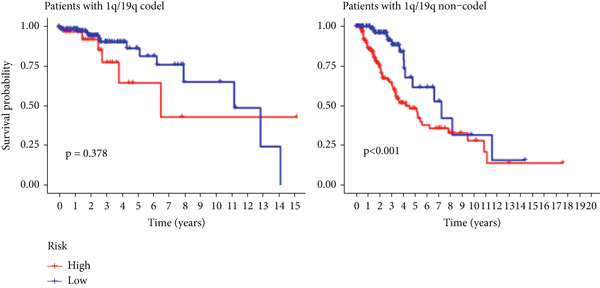
(f)

**Figure:**
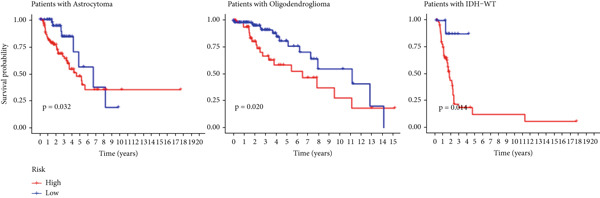
(g)

### 3.5. Mutation Between High‐Risk Group and Low‐Risk Group

Our examination of the association between risk score and tumor mutation load (TMB) and the variance in TMB across different risk groups (Figure 8b) revealed that TMB was higher in the high‐risk group (Figure 8a). The most commonly altered genes were IDH1, TP53, and ATRX in the high‐risk and low‐risk groups, respectively (Figure 8c,d). The high‐risk group, however, had more mutations in other genes and fewer IDH mutations. In order to compare the specific traits of gene mutation between high‐risk and low‐risk populations, we made two waterfall plots.

Figure 8Mutation analysis based on risk score model. (a) Differences in tumor mutational load (TMB) in high‐flying risk score groups. (b) Correlation of risk score and TMB. (c, d) Waterfall plots summarizing the mutations in high‐ and low‐risk patients.

**Figure:**
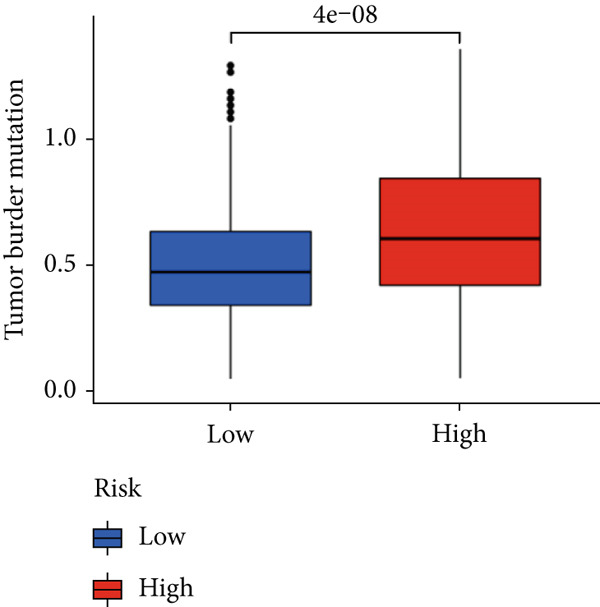
(a)

**Figure:**
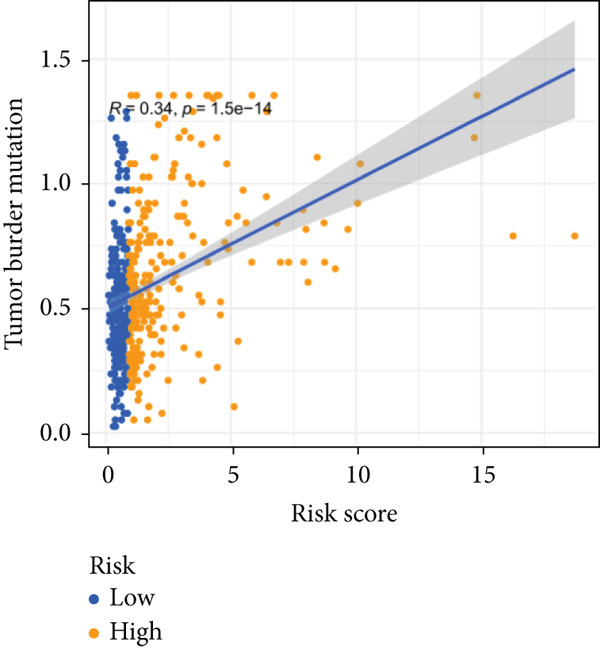
(b)

**Figure:**
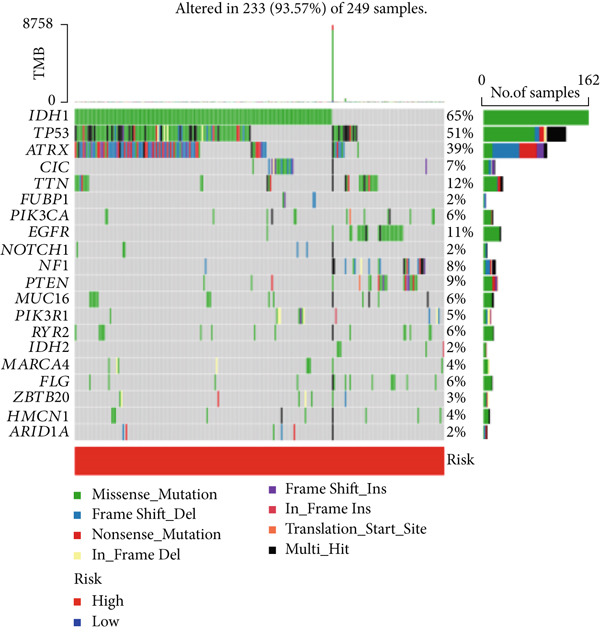
(c)

**Figure:**
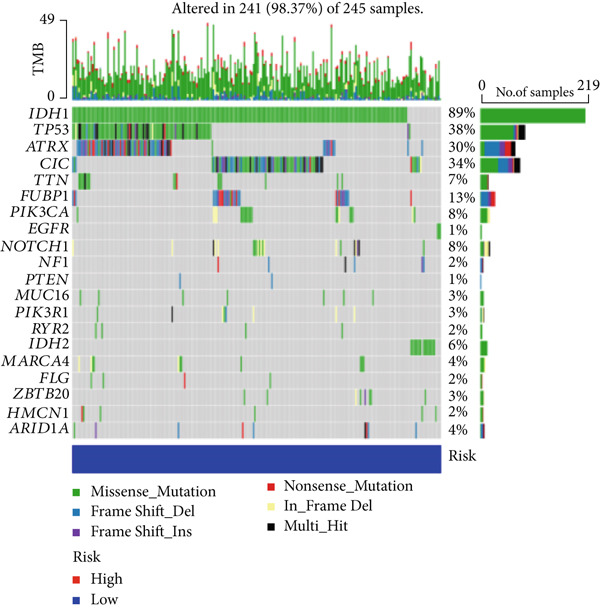
(d)

### 3.6. Drug Sensitivity and Gene Set Enrichment Analysis

We looked into the potential sensitivity of clinical agents in the high‐risk and low‐risk groups and screened a number of potential chemotherapeutic drugs for the treatment of gliomas (Figure 9a,b), which suggested that patients with higher risk scores might be more sensitive. The high‐risk group had higher levels of B cell receptors, actin cytoskeleton modulation, the Jak/Stat signaling system, and cancer pathways (Figure 9c). IVM can prevent GLUT4 from activating the JAK/STAT signaling pathway, which would otherwise cause glycolysis to be inhibited and glioma cells to die more quickly from autophagy [19]. The phosphatidylinositol signaling system, long‐term potentiation, neuroactive ligand‐receptor interactions, and calcium signaling pathways, on the other hand, were more prevalent in the low‐risk group. Then, using GSVA software, we concentrated on the distinct enrichment of the KEGG pathway between high and low‐risk subgroups given the obvious variations between the set’s various risk subgroups (Figure 9d). The high‐risk group with the poorest prognosis was mostly linked to immune‐related illness pathways as well as intercellular communication.

Figure 9Drug sensitivity analysis and enrichment analysis in different risk score groups. IC50 values were calculated for patients in the high‐ and low‐risk groups based on Lapatinib (a) and Erlotinib (b) to assess the sensitivity of chemotherapeutic agents. GSEA (c) and GSVA (d) analysis focused on the differential enrichment of KEGG pathways.

**Figure:**
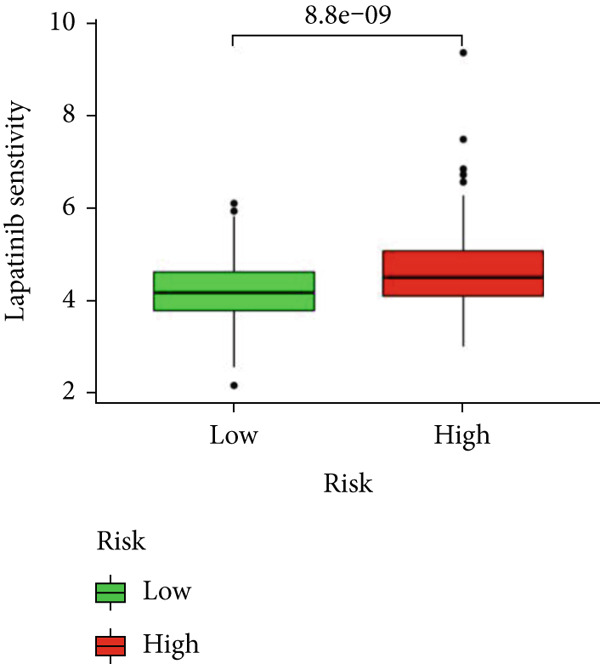
(a)

**Figure:**
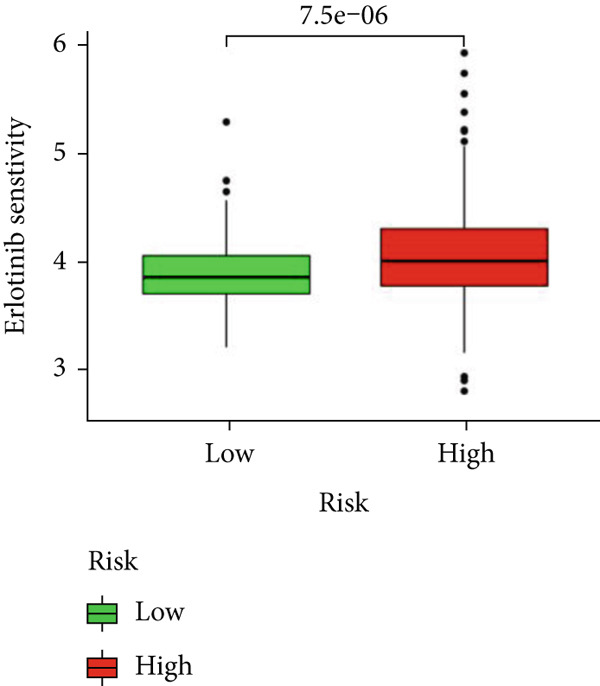
(b)

**Figure:**
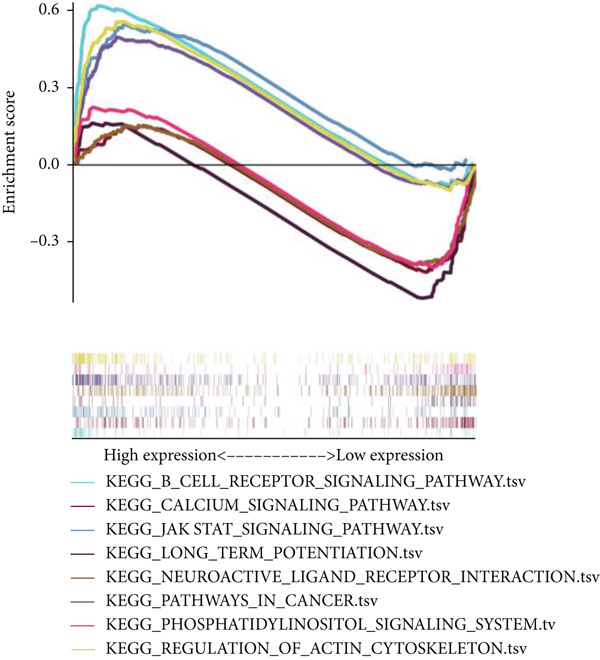
(c)

**Figure:**
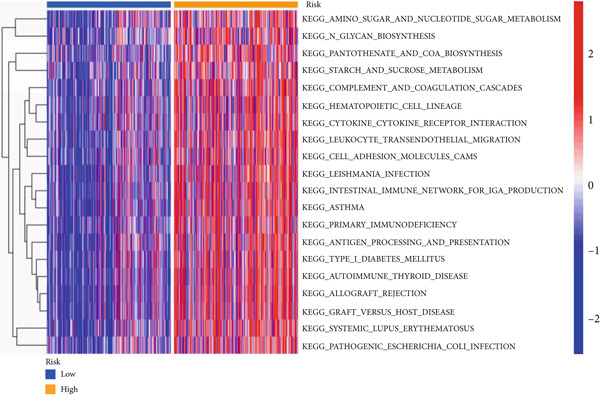
(d)

### 3.7. Validation of Model Genes

Next, we examined the association between model genes and survival in high‐ and low‐risk scoring groups. The results showed that hsa‐miR‐10b‐5p, hsa‐miR‐27a‐3p, hsa‐miR‐30a‐5p, hsa‐miR‐409‐3p, hsa‐miR‐424‐5p, hsa‐miR‐93‐5p, and hsa‐miR‐128‐3p had a significant positive association with survival (Figures 10a, 10b, 10c, 10d, 10e, 10f, and 10g). Correlation analysis showed a significant association between these seven miRNAs (Figure 10h). Finally, we compared the expression differences of these seven miRNAs in LGG and normal tissues in the GEO cohort (Figure 10i). Probably due to the small sample size, the differences in expression of all miRNAs were statistically significant except for hsa‐miR‐93‐5p. A review of the current literature on microRNAs reveals a notable gap in research concerning hsa‐miR‐93‐5p in the context of gliomas. Consequently, we selected this microRNA for functional validation. q‐PCR analysis indicated that LN299 cells exhibited the highest expression of hsa‐miR‐93‐5p, leading to its targeted inhibition (Figure 10j). Results demonstrated that suppressing hsa‐miR‐93‐5p expression significantly impaired LN299 cell proliferation (Figure 10k,l). These findings suggest that hsa‐miR‐93‐5p may serve as a potential target for future glioma‐specific therapies.

Figure 10Validation of seven CRMs. (a) hsa‐miR‐10b‐5p, (b) hsa‐miR‐27a‐3p, (c) hsa‐miR‐30a‐5p, (d) hsa‐miR‐93‐5p, (e) hsa‐miR‐424‐5p, (f) hsa‐miR‐409‐3p, and (g) hsa‐miR‐128‐3p in the TCGA predictive analysis of high and low risk score groups in the cohort. (h) Correlation analysis of seven miRNAs. (i) Validation of CRMs expression in the GEO cohort.  ^∗^ *p* < 0.05,  ^∗∗^ *p* < 0.01,  ^∗∗∗^ *p* < 0.001. (j) RT‐PCR was employed to assess the expression levels of hsa‐miR‐93‐5p in various glioma cell lines. (k) The efficacy of hsa‐miR‐93‐5p inhibitors was evaluated. (l) CCK8 assays were conducted to examine differences in LN299 cells with varying levels of hsa‐miR‐93‐5p expression.

**Figure:**
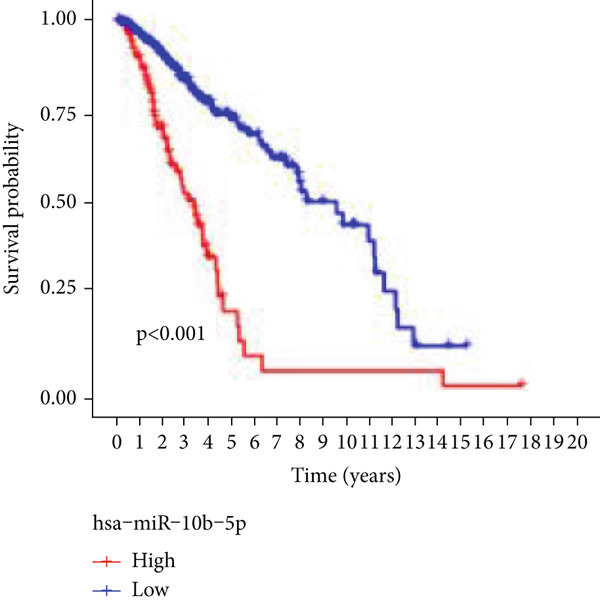
(a)

**Figure:**
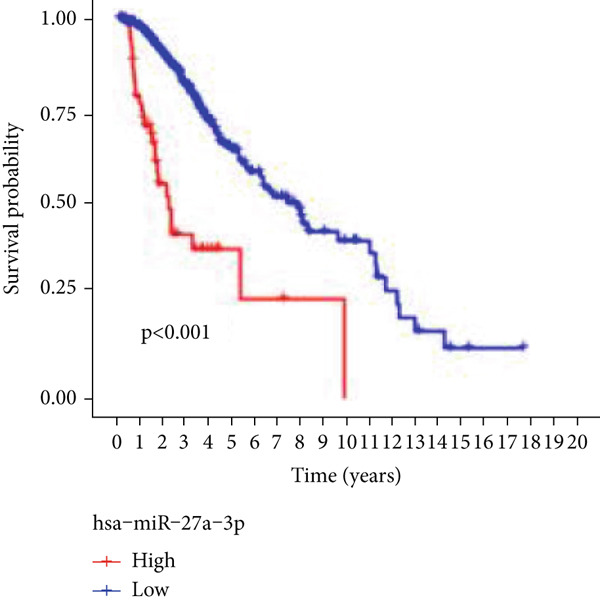
(b)

**Figure:**
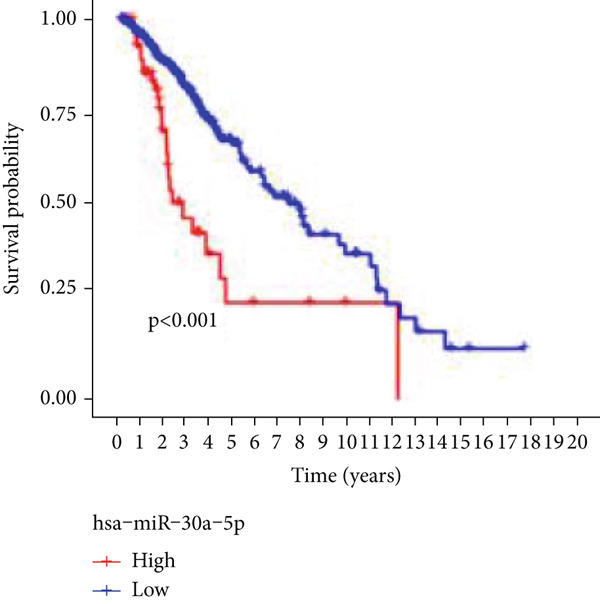
(c)

**Figure:**
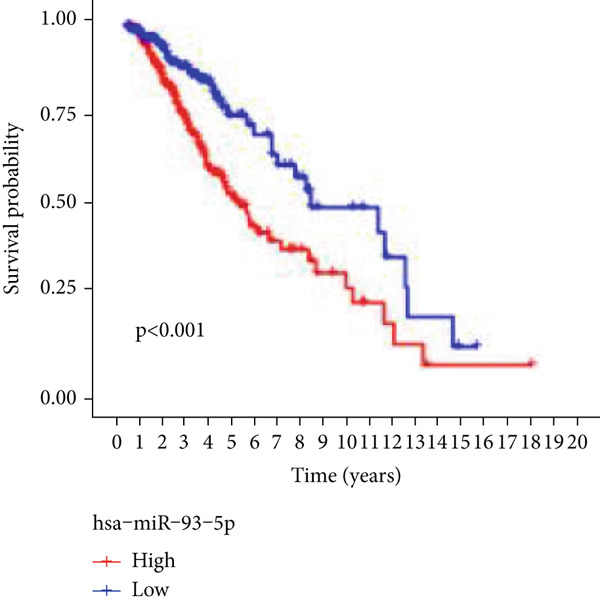
(d)

**Figure:**
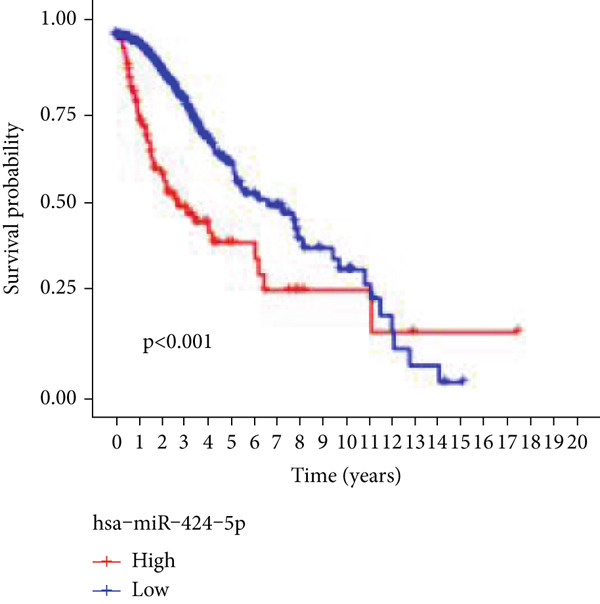
(e)

**Figure:**
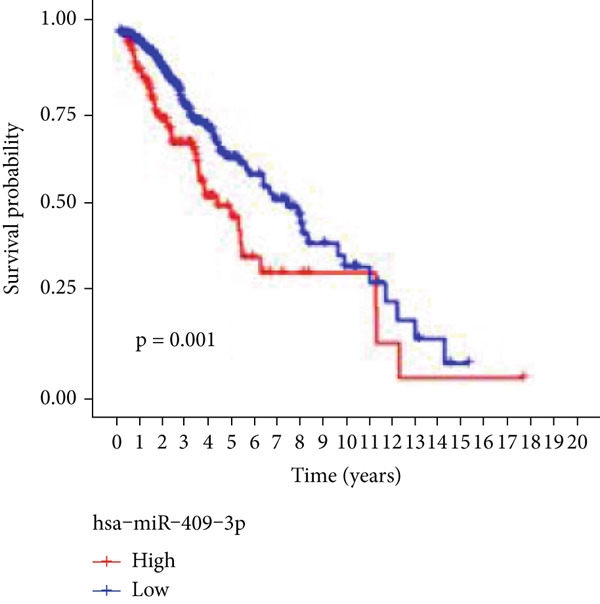
(f)

**Figure:**
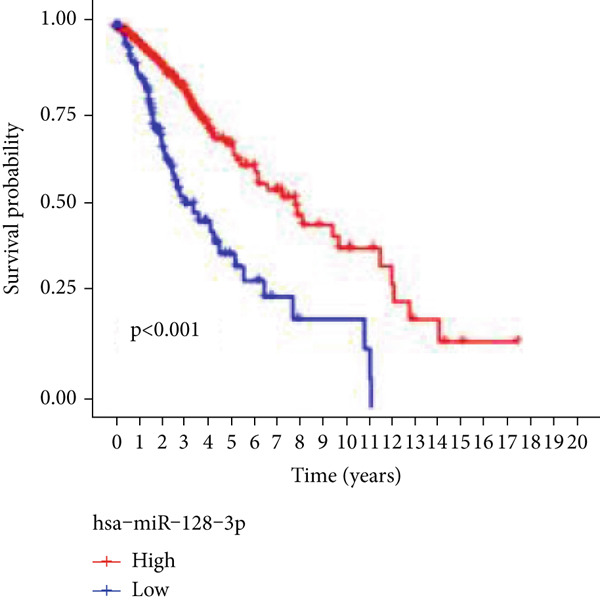
(g)

**Figure:**
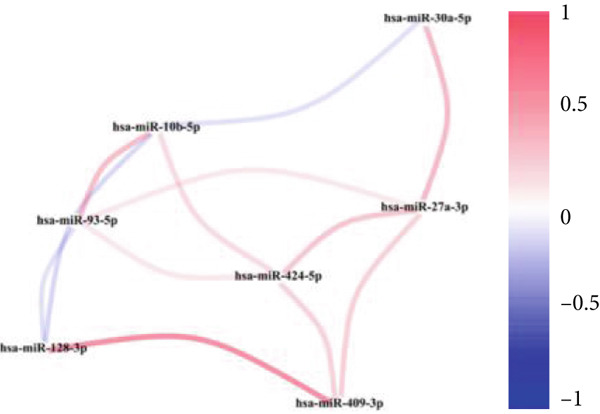
(h)

**Figure:**
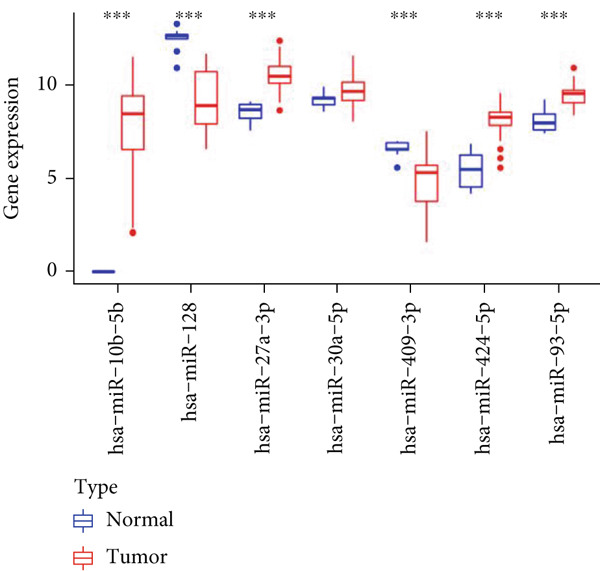
(i)

**Figure:**
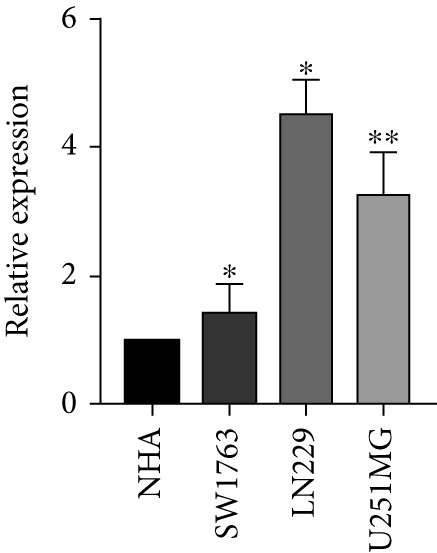
(j)

**Figure:**
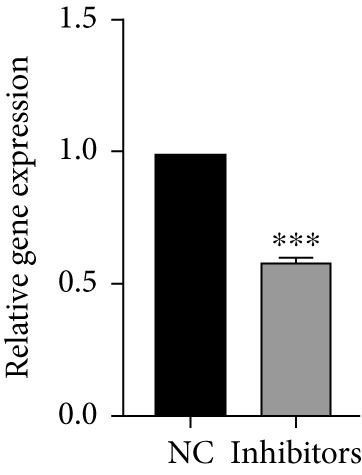
(k)

**Figure:**
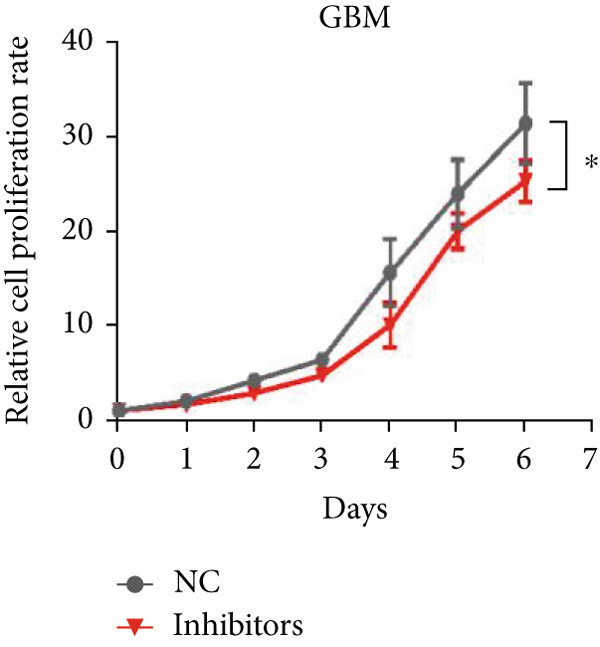
(l)

## 4. Discussion

One of the characteristics of glioma is its great spatial and temporal heterogeneity, making the disease difficult to diagnose and identify genetic mutations [20]. Compared with IDH mutant gliomas, IDH‐wt gliomas usually have a worse prognosis [21]. Previous studies have reported that 1p/19q co‐deletion can affect the pathological behavior of gliomas [22]. Moreover, existing literature indicated that ferroptosis could affect the prognosis of patients with glioma and other malignancies [23–25]. The metabolic functions of life, such as mitochondrial respiration, iron absorption, and antioxidant activities, both need copper, an important mineral ingredient for all living things [26]. In cells characterized by glutathione deficiency and environmental copper enrichment, disruption of the internal environment drives oxidative stress, interfering with bidirectional communication between neurons and astrocytes, ultimately leading to various forms of brain disease [27]. In addition, some data suggested that the cupric oxide compound may induce glioma cell death through autophagy and apoptosis [28]. Also, copper ions have a signaling role, and significant alterations in copper levels have been found in various tumor tissues. Copper may participate in tumors’ etiology, severity, and progression [29]. Furthermore, controlling intracellular copper levels may be used to selectively kill tumor cells [30]. Therefore, the study of Cu is of great importance, and it is also likely to replace ferroptosis as a potential target to inhibit cancer development. Currently, many models for prognostic features of tumors based on ferroptosis have been reported in glioma [31, 32]. However, CRMs prognostic signature models have not been studied in tumors.

On the basis of the TCGA dataset and the Targetscan database, we initially identified 157 CRMs in our study that had prognostic value. Seven miRNAs related to cuprotosis were modeled in the end. Our risk assessment model fared better in predicting LGG prognosis than conventional clinicopathological characteristics, indicating that the CRM signature may accurately predict the prognosis of patients with LGG. Next, we looked at the involvement of immune infiltrating cells in the tumor microenvironment and immune checkpoint inhibitors (ICIs) in glioma prognosis. We discovered that the high‐risk group with poor prognosis had increased immune cell abundance and immune checkpoint gene expression. Additionally, the high‐risk group had a lower IDH mutation rate but a greater tumor mutational burden. Finally, we tested its capacity for risk categorization using an external validation cohort.

In this investigation, we constructed a highly sophisticated prognostic model that incorporates six high‐risk genes. The expression profiles of these miRNA molecules within the tumor microenvironment are intricately correlated with the malignant progression of glioma and patient outcomes, thereby corroborating extant findings. From the standpoint of tumor metabolism, these miRNA molecules orchestrate the metabolic network of tumor cells at multiple strata, furnishing the material and energy resources requisite for the propagation of malignant tumors. Certain high‐risk miRNAs can enhance the glycolytic flux of tumor cells by modulating the expression of glycolysis‐associated genes, thereby augmenting lactate production. This metabolic adaptation enables tumor cells to sustain their energy demands and rapid proliferation in hypoxic microenvironments. For instance, the overexpression of hsa‐miR‐27a‐3p markedly suppresses the expression of FANCD2 and CD44, consequently inhibiting the formation and growth of gliomas in murine models [33]. The expression of miR‐30a‐5p is significantly upregulated and exhibits a positive correlation with tumor malignancy, implicating its pivotal role in tumorigenesis [34]. MiR‐409‐3p facilitates the proliferation of glioma cells and expedites cell cycle progression by precisely regulating the expression of PDK1 through the ceRNA network, thereby driving tumorous expansion [35]. Concomitantly, the FAM87A gene downregulates the expression of PPM1H by engaging in competitive binding with miR‐424‐5p, thereby exerting a suppressive effect on cell proliferation, migration, and invasion [36]. Among the low‐risk genes, miR‐128‐3p promotes cell growth by upregulating ITGA5 expression in glioma cells and activating the FAK signaling cascade Among the low‐risk genes, miR‐128‐3p promotes cell growth by upregulating ITGA5 expression in glioma cells and activating the FAK signaling cascade [37]. These findings not only enhance our understanding of the mechanisms governing miRNA’s role in glioma but also provide a robust theoretical foundation and potential molecular targets for the development of innovative miRNA‐based cancer therapies.

The risk model was more accurately predicted by multivariate Cox regression analysis than by clinicopathological traits. The risk model performed better than other clinical features in predicting the prognosis of LGGs, as evidenced by the fact that the AUC of the risk score was higher than that of the clinical features. Thus, our results show that in patients with LGG, the CRM signature accurately predicts OS.

An increasing number of aberrant signaling pathways are being uncovered and studied in gliomas. Some of these aberrant signaling molecules can be used to identify new therapies, such as the Wnt target signaling pathway [38] and the PI3K/AKT pathway [39]. In this study, we identified various immunoregulatory mechanisms in high‐risk and low‐risk categories, which may help determine how patients with glioma are treated in the future. Furthermore, the variable antitumor immunity between high‐risk and low‐risk patients may also assist determine how patients with glioma are treated in the future. Clinical research is currently being done in the area of glioma immunotherapy [40]. However, based on several potential biomarkers of response to immune checkpoints, only a small proportion of patients with glioma may benefit from immune checkpoint inhibition [41]. According to several earlier research, the activation of T lymphocytes results in the death of tumor cells, which is directly related to the development of ferroptosis and antitumor immunity [42]. As a result, we think that combining cuprotosis and ICI will likely increase their antitumor actions synergistically. In our investigation, high‐risk groups expressed the majority of ICIs at higher levels than low‐risk groups. This implies that the immune checkpoint blockade immunotherapy may be guided by the seven CRM signature, which may be utilized to anticipate the amounts of ICI expression. The combination of ICIs with cuprotosis inducers may encourage the demise of malignant glioma cells in patients with high‐risk LGG, improving overall prognosis.

The study has several restrictions. First of all, since bioinformatics analysis was used to acquire all of these conclusions, further experimental confirmation is required. Second, our work created a biomarker for the miRNA related with genetic cuprotosis, and large‐scale clinical studies are required to corroborate our results.

## Ethics Statement

The authors have nothing to report.

## Consent

The authors have nothing to report.

## Disclosure

All authors reviewed and approved the submitted manuscript.

## Conflicts of Interest

The authors declare no conflicts of interest.

## Author Contributions

Zhaoliang Xue designed this study. Bioinformatics analyses were performed by Zhaoliang Xue and Zhengfei Song. All authors contributed to the experiments and the analysis of data. The first draft of the manuscript was written by Zhaoliang Xue, Lianjie Mo, and Shuxu Yang.

## Funding

No funding was received for this manuscript.

## Supporting Information

Additional supporting information can be found online in the Supporting Information section.

## Supporting information


**Supporting Information 1** Table S1: Sample information of the database.

**Supporting Information 2** Table S2: MicroRNAs corresponding to copper death genes.

**Supporting Information 3** Table S3: Modeling gene coefficient.

## Data Availability

The datasets analyzed for this study were obtained from the UCSC Xena website (http://xena.ucsc.edu/) and CGGA dataset (http://www.cgga.org.cn/). The data generated in the present study may be requested from the corresponding author.
